# Effect of resin thickness, and curing time on the micro-hardness of bulk-fill resin composites

**DOI:** 10.4317/jced.52536

**Published:** 2015-12-01

**Authors:** Shaymaa M. Nagi, Lamiaa M. Moharam, Mohamed H. Zaazou

**Affiliations:** 1 Restorative and Dental Materials Research department, National Research centre, Giza. Egypt

## Abstract

**Background:**

Bulk-fill resin composite has been introduced, their manufacturers claimed that they can be applied in bulks of 4mm, without necessitating a prolonged curing time, or a light curing unit with increased irradiance. Thus this study was conducted to evaluate the effect of resin thickness, and curing time on the micro-hardness of two bulk -fill resin composites; Tetric Evo-Ceram [TE], and X-trafil [XF].

**Material and Methods:**

120 cylindrical specimens were prepared, and divided into 24 groups (n=5/group), representing the two bulk-fill resin composites, three different material thicknesses (2, 3 and 4 mm) and the four curing times used in the study (10, 20, 40, and 60 seconds). The specimens were light-cured from the top surface only. Specimens were stored in light proof containers in complete darkness at 37°C for 24 hours. Micro-hardness test was conducted on both top and bottom surfaces using Vickers micro-hardness tester with 500 g load and a dwell time of 15 seconds. Data were statistically analyzed by Four-way ANOVA of Variance. The significance level was set at *P* ≤ 0.05. Pearson Correlation used to determine significant correlations between mean micro-hardness (top) and (bottom) surfaces.

**Results:**

Four way-ANOVA shows that different tested materials produce a statistically significant effect on mean micro-hardness (VHN) at *p*≤0.001, while thickness, curing time, and surface revealed statistically non significant effect on mean micro-hardness (VHN) at *p*≥0.05. [XF] (92.01±3.15 VHN) showed statistically significant higher mean micro-hardness than [TE] (54.13±4.96 VHN). Pearson Correlation revealed that there was a significant direct correlation between micro-hardness (bottom) and mean micro-hardness (top) (mm), r = 0.985, *p* (2-tailed) ≤0.001.

**Conclusions:**

Within the limitations of this study, the bulk-fill resin composites used in this study can be placed and cured properly in the 4 mm bulk.

** Key words:**Bulk-fill resin composite, micro-hardness, thickness, curing time.

## Introduction

Resin composites are the widely used esthetic restorative materials. So manufactures have always to improve them in terms of the chemical composition and filler reinforcements. Dental composite restorations have a major drawback regarding the degree of cure, which is proportional to the amount of light they are exposed. So, they polymerize to a certain depth which varies with the penetration of a light beam in the bulk material. This extent of cure has been termed (depth of cure) and has significant influence on both physical and biological properties of restorations. The depth of cure is the depth to which the light is able to harden the material (1). So that layering technique for resin composite has been a central point in teaching direct resin composite restorations, to ensure their curing.

Recently, many clinicians have shown the preference for time saving restorative procedures for posterior resin applications. A new category of resin composites, a bulk-fill resin composite, has been introduced over the past few years. According to the manufacturers, these materials can be applied in bulks of 4mm, without necessitating a prolonged curing time, or a light curing unit with increased irradiance, thereby skipping the time-consuming layering process. Although the manufacturers recommend bulk-filling of these materials up to 4 mm, many clinicians suspect that the depth of cure and mechanical properties might not be suitable for clinical use (2). Hardness measurements of the bottom surface can be used to evaluate the depth of cure for resin composites (2-5).

There are few reports on of the effect of resin thickness, and the curing time of these bulk-fill resin composites on the micro-hardness. Therefore, this study investigated the effects of the resin thickness, and curing time on the micro-hardness of two of these bulk-fill resin composites.

## Material and Methods

-Study design and specimen grouping.

A total of 120 cylindrical specimens were prepared and then equally divided into 24 groups (n=5/group), representing the two bulk-fill resin composites used in the study (Tetric Evo-Ceram [TE] and X-trafil [XF]), the three different material thicknesses (2, 3 and 4 mm) and finally the four curing times used in the study (10, 20, 40, and 60 seconds (sec.)). The materials brand name, manufacturers, and their composition are listed in  Table 1.

**Table 1:**
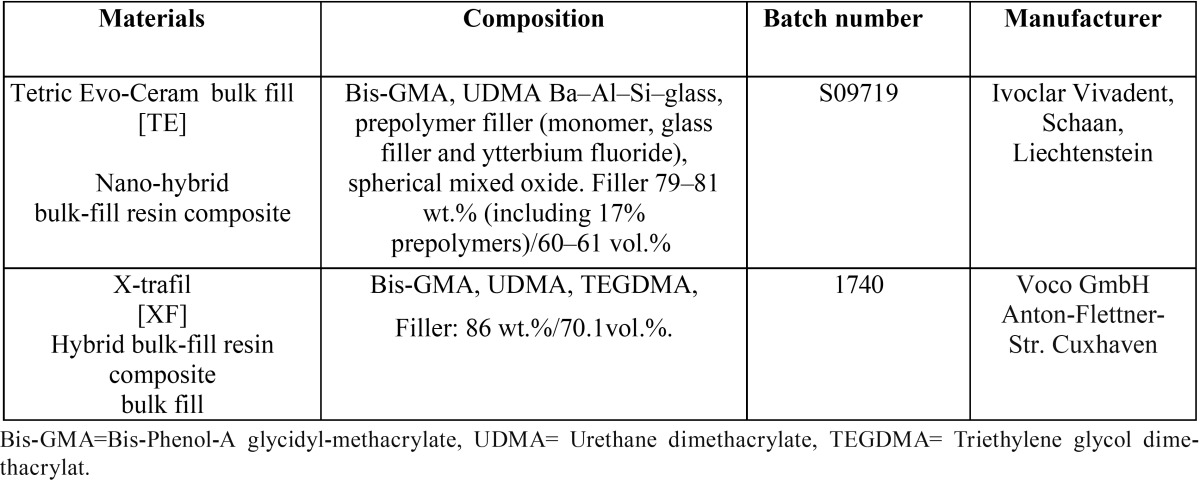
Materials brand name, composition, and manufacturers.

-Specimens preparation:

Sectional Teflon molds of 6 mm diameter and different thickness (2 mm, 3 mm and 4 mm) were used to prepare the specimens. The molds were first mounted on the top of a microscope slide and a Mylar strip, and then the mold was filled in bulk with one of the two bulk-fill resin composites. The top side of the mold was covered with a second Mylar strip to prevent oxygen inhibition. A glass microscope slide with a load of 1 kg was applied for 30 seconds to ensure consistent packing of the specimens (6). The load and microscope slide were then removed. The specimens were light-cured from the top surface only using LED Elipar S10 light curing unit (Elipar S10, 3M ESPE; USA) with an output ≥ 800 mW/cm2 for 10, 20, 40, or 60 sec. The light curing tip was kept centered and in direct contact with the second Mylar strip. The power density of light curing unit was assessed using a hand-held radiometer (Curing Radiometer, Demetron, Danbury, CT, USA). After light-curing, the cylindrical specimens were pushed out of the mold and the uncured resin composite material was removed with a plastic spatula (3). The top surfaces of the specimens were identified with an indelible mark. Specimens were stored in light proof containers before the tests were conducted, in complete darkness at 37°C for 24 hours to prevent ambient light from causing additional post light-curing polymerization (7,8). 

-Micro-hardness testing.

The prepared specimens were tested for their micro-hardness using Vickers micro-hardness tester (Nexsus 4000/60, INNOVATEST, Netherlands, Europe). Six randomized indentations (3 on both the top and bottom surfaces) were made with 500 g load and a dwell time of 15 seconds (6,8). For randomization, specimens were arbitrarily rotated before indentations. Calculations were made using computer software (Hardness-Course Vickers/ Brinell/ Rockwell copy right IBS 2012 version 10.4.4).

-Statistical analysis.

Data were presented as mean, standard deviation (SD) and standard error (SE) values. Data were explored for normality using D’Agostino-Pearson test for Normal distribution. Four-way ANOVA was used to study the effect of different tested restorative materials, thickness, surface and curing time on mean micro-hardness. Tukey’s post-hoc test was used for pair-wise comparison between the means when ANOVA test is significant. Independent t-test had been used to compare between different tested resin materials and surface. One way-ANOVA have been used to study the effect of thickness and curing time on mean micro-hardness followed by Tukey’s post-hoc test was used for pair-wise comparison between the means when ANOVA test is significant. The significance level was set at *P* ≤ 0.05.

Pearson Correlation used to determine significant correlations between mean micro-hardness (top) and (Bottom).

Statistical analysis was performed with IBM® SPSS® (SPSS Inc., IBM Corporation, NY, USA) Statistics Version 22 for Windows.

## Results

Four way-ANOVA shows that different tested materials produce a statistically significant effect on mean micro-hardness (VHN) at *p*≤0.001. On the other hand; thickness, curing time, and surface revealed statistically non significant effect on mean micro-hardness (VHN) at *p*≥0.05.

Comparing the two tested resin composite materials, [XF] (92.01±3.15 VHN) showed statistically significant higher mean micro-hardness than [TE] (54.13±4.96 VHN).

Mean and standard deviation (SD) for the micro-hardness (VHN) for the effect of different thickness and curing time tested within each group were presented in  Table 2.

**Table 2:**
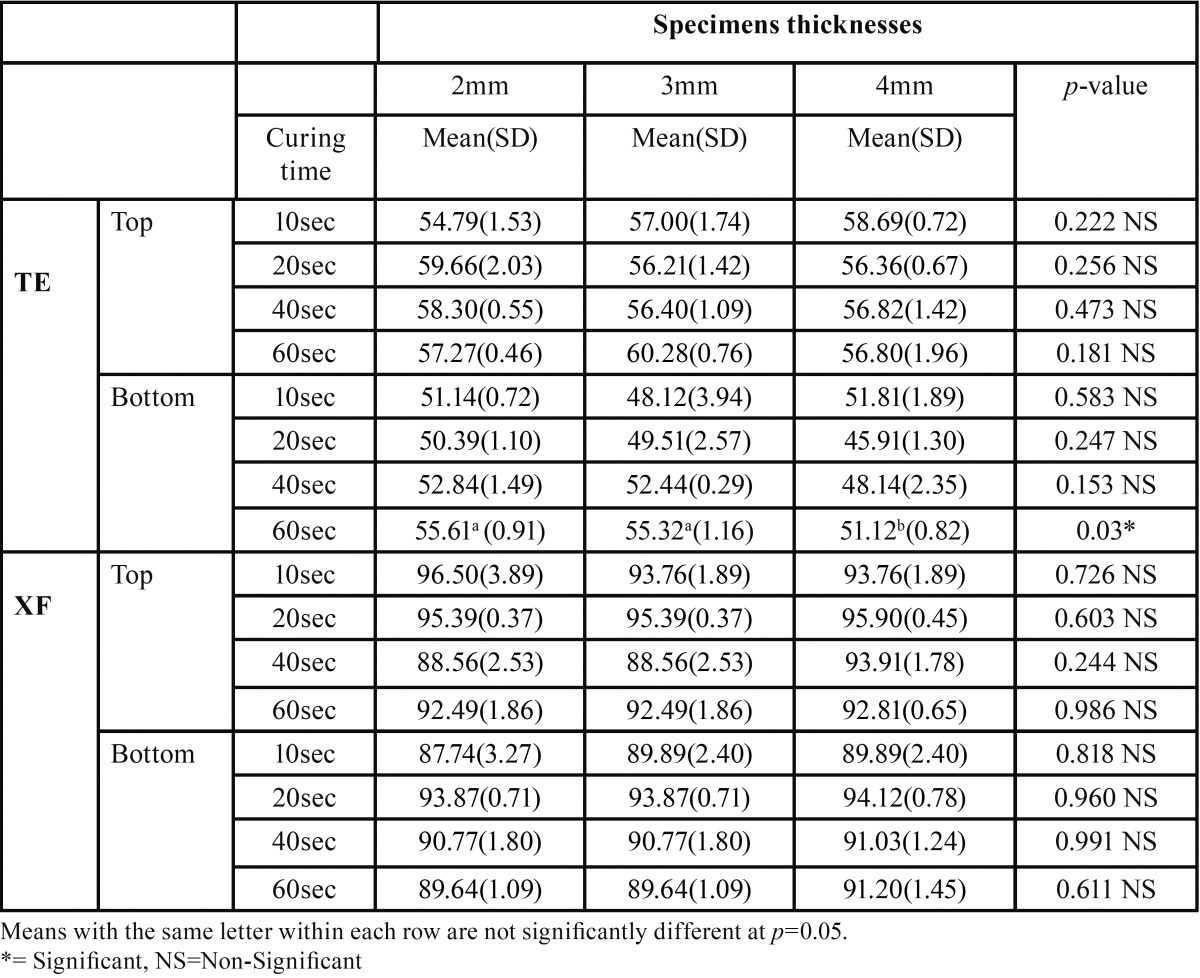
Mean and standard deviation (SD) for the mean micro-hardness (VHN) for the different tested resin composite thickness and curing time within each group.

Results revealed that there was no statistically significant effect of the different material thickness (2, 3, 4 mm) on the mean micro-hardness of both top and bottom surfaces. Except for TE, 4 mm bottom surface showed statistically significant lower mean micro-hardness compared to 2 and 3mm thickness bottom surfaces at *p*=0.03, when specimens cured for 60 seconds. 

On the other hand, results revealed that there was no statistically significant effect of the different curing time (10, 20, 40,and 60 seconds) on the mean micro-hardness of both top and bottom surfaces. Except for TE, the bottom surface of 2 mm thickness specimens cured for 60 sec. was statistically significantly lower than the bottom surface of specimens cured for 10, 20 and 40 seconds, at *p*=0.04.

**Figure 1:**
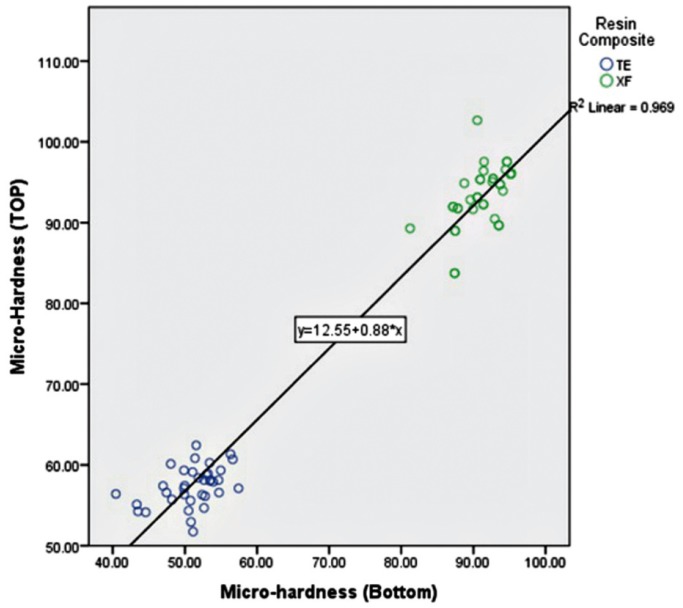
Scatterplot of mean micro-hardness (top) and (bottom) surfaces for tested materials.

Results of Pearson Correlation for the correlation between mean micro-hardness (VHN) for top and bottom surfaces revealed that there was a significant direct correlation between micro-hardness (bottom) and mean micro-hardness (top) (mm), r = 0.985, *p* (2-tailed) ≤0.001, (Fig. 1).

## Discussion

In the current study XF showed statistically significant higher mean micro-hardness than TE, at all tested curing time (10, 20, 40, 60 seconds), and different specimens thickness (2, 3, 4mm). This result might be due to the different chemical composition of the two tested bulk- fill resin composites. It seems that manufacturers had followed different strategies to increase the depth of cure in these bulk fill resin composites. In XF the manufacturer increased the filler size and filler content. Several studies (9,10) confirm that the high inorganic filler amount of XF was directly reflected in the measured mechanical properties and unexpectedly, the depth of cure at a given exposure condition. This is however in accordance with measurements of the transmitted light (360-540 nm wavelength) through the specimens of different thicknesses (2, 4, and 6 mm), emphasizing a higher translucency for XF compared with TE (9).

In general, the translucency of all resin based composite (RBCs) was shown to increase during irradiation as the polymerization process progresses from initiation to polymer network formation. The amount of light transmitted through a RBC is dependent on the amount of scattered and absorbed light (11). As dental RBCs consist of heterogeneous substances, resin and fillers, the passing light is scattered at the resin-filler interface, due to differences in the refractive indices of the individual compounds. Light transmittance in dental RBCs was shown to decrease with increased filler content and for irregular filler shape (12). This is due to the increase of specific surface between fillers and resin. Consequently, the specific surface between fillers and organic matrix is lowered, thus reducing light scattering. The explanation for the high translucency in XF despite a high filler amount must be searched in the increased filler size and a potentially improved matching between the refractive indices of filler particles and the resin matrix (9,13,14). The last essentially determines how light is scattering within a material (15). In addition, generally in resin composite materials degree of conversion is highly correlated to the hardness of the material (16), explained by the higher density achieved in the densely compacted cross-network of dental resin composites. However, this is not the only factor influencing the hardness of dental composite resins, and fillers are recognized as more influential. The exceptions are microfilled composites with prepolymerized filler particles and higher amount of organic matrix (17), similar to TE material in this study. Although it contains nano-filler particles, which characterizes it as a nanohybrid composite resin, it also contains prepolymerized resin fillers, which are consisted of fillers embedded in resin, polymerized and milled to obtain a desired particle size. Therefore, prepolymerized fillers never achieve as high micro-hardness values as the composites without prepolymerized particles (18), which is in agreement with our results.

The positive effect of different strategies that manufacturers have followed to increase the depth of cure can be emphasized in this study, since the VHN measured in both tested bulk-fill resin composites was constant at all incremental thicknesses (2, 3, 4 mm).

As discussed before, in XF the manufacturer increased the filler size. Consequently, the specific surface between fillers and organic matrix is lowered, thus reducing light scattering. A different way to enhance depth of cure was followed in TE by introducing an additional photo-initiator (Ivocerin), which is considered to be more effective than CQ (19,20). Additionally, the shape of Tetric EvoCeram Bulk Fill fillers is approaching round-shaped fillers, which were shown to positively influence the translucency (20).

Several studies (2-5) have used hardness measurements performed on the top and bottom surface of light-cured resin composite specimens to define the depth of cure. In the present study; no significant reduction in the bottom to top micro-hardness values of the materials investigated (XF, and TE) at the different tested specimens thickness was evident. Moreover both tested bulk fill resin composite materials reached VHN bottom to top ratio above 80% when cured according to the manufacturers (10 seconds). It has been reported that resin-based filling materials should exhibit a minimum of 80% bottom/ top hardness percentage when cured in a 2mm increment in order to be considered as adequately polymerized (4). Accordingly, in the current study, a similar percentage at 4 mm depth was considered acceptable curing, and above 90% was considered high curing efficiency. The results revealed that both investigated bulk fill materials exhibited high curing at the deepest portion of a 4mm increment (bottom/top% ≥80%). This is in agreement with comparable studies that showed that bulk-fill materials met the requirements stipulated in the ISO 4049 specification, even with a light curing time as short as 10 seconds (3,21,22).

## Conclusion

Within the limitations of this study, the bulk-fill resin composites used in this study can be placed and cured properly in the 4 mm bulk.

A short curing time 10 seconds was enough to reach HV (bottom to top) ratio >80 % when both tested bulk fill RBCs are placed in 4 mm bulks.
